# Behavioral synchronization of two individuals during cooperative interaction: the role of the mentalization ability as measured by the reading the mind in the eyes test

**DOI:** 10.1186/s40359-025-02457-x

**Published:** 2025-02-16

**Authors:** Brigitta Kakuszi, István Bitter, Pál Czobor

**Affiliations:** Department of Psychiatry and Psychotherapy, Semmelweis University, Budapest, Hungary

**Keywords:** Mentalization, Theory of mind, Cooperative interaction, Reading the mind in the eyes test, Two-subject design, Synchronous behavior

## Abstract

**Background:**

Interpersonally synchronized behaviors play a fundamental role in social interactions. An important prereqvisite for behavioral synchronization is the mentalization ability of the individuals involved in the interaction. While the Reading the Mind in the Eyes Test (RMET) is widely applied to measure mentalization, most studies of RMET used single-subject designs, which lack ecological validity and have limited generalizability for real, on-going, reciprocal social interactions. Using a two-subject design, our objectives were to examine (i) the predictive validity of RMET for the synchronous behavior of two interacting individuals during a reaction time task; (ii) the association of age-related RMET performance with interpersonal coordination; and (iii) the influence of various potentially important covariates on the association, including psychological distress, basic personality domains such as impulsive responding, and lack of attention.

**Methods:**

We investigated 24 dyads, using 48 subjects (mean age 32.9 [SD = 12.8] years). Synchronous behavior was indexed by the correlation of participants’ responses in terms of reaction times (RTs) to visual stimuli. We used the correct recognition rate from the RMET as a measure of mentalization ability.

**Results:**

Our results indicated that the synchronization of the behavioral responses (i.e., RTs) between the interacting pairs and their mentalization ability, as measured by the RMET, showed significant association. Specifically, with good performance (100% correct recognition), the behavioral response synchronization was significantly (F = 153.34, *p* < 0.0001) higher (Pearson correlation, *r* = 0.41) than with poor performance (50% correct recognition, *r* = 0.25). Higher age was inversely related to behavioral synchronization, with no interaction (*p* > 0.1) with RMET performance). The inclusion of additional covariates (e.g., measures for psychological distress and basic personality domains) in the analyses did not alter the significance of the association between RMET performance and behavioral synchronization (*p* < 0.05, after adjustment).

**Conclusions:**

Our results confirm the utility of RMET, as a measure of mentalization, to predict participants’ ability to achieve behavioral synchronization during cooperative interaction. These results may facilitate research into mental disorders, where problems with social interactions play a role in symptom presentation. For this purpose, future studies with larger sample size should examine whether our findings can be dimensionally extrapolated to patients with such disorders.

**Supplementary Information:**

The online version contains supplementary material available at 10.1186/s40359-025-02457-x.

## Introduction

Interpersonally synchronized behaviors play a fundamental role in social interactions. An important prerequisite of successful behavioral synchronization is the “Theory of Mind”(TOM) ability of the people who are involved in the interaction [1]. TOM reflects the ability to attribute mental states to ourselves and others, which serves as one of the foundational elements for social interaction, as it underpins the capacity to predict and interpret the behavior of others. A well-functioning “mind-reading ability” can confer a competitive advantage in real life situations through the facilitation of interpersonal relationships [2]. Paal and Bereczkei’s study [3] demonstrated that the degree of cooperative predisposition, as measured by the social cooperation subscale of the Cloninger Temperament and Character Inventory [4] is dependent on the TOM ability. The better the individuals’ ability to read an interacting person’s mind, the greater their willingness is to collaborate with others [3].

It is essential to recognize that Theory of Mind (ToM) ability intersects with the broader concept of mentalization. According to Fonagy’s influential theory, mentalization encompasses four dimensions, spanning the poles of Affective vs. Cognitive, Other-Oriented vs. Self-Oriented, External vs. Internal, and Automatic vs. Controlled [5]. Theory of Mind can be viewed as a foundational ability that contributes to the broader and more relational process of mentalization, which builds on a well-developed ToM while also encompassing aspects that go beyond its scope. Among several tools available to delineate TOM, the “Reading the Mind in the Eyes Test” (RMET), developed by Baron-Cohen and co-workers [6, 7] is a widely used one to measure mentalization and empathy as well as social sensitivity [8, 9]. While the test emphasizes external mentalizing, it also engages implicit mentalizing, as it involves automatic, intuitive judgments based on limited information [6, 7, 9–12].

The RMET was designed to test how well the interacting persons can put themselves into the mind of the other persons, and ‘‘tune in’’ to their mental state. The test has yielded promising results for the characterization of individuals with TOM impairment, including people with schizophrenia and autism spectrum disorder (ASD) [13].

RMET’s potential clinical utility was supported by the poor test performance in ASD [7], a group that manifests TOM impairments [14]. A web-based study concluded that the performance on the RMET is „unduly influenced” by various demographic and socio-cultural factors [15]. In particular, this study found that females, and more highly educated, non-Hispanic, and White/Caucasian individuals performed best. In contrast to most other studies [16–18], the study reported that performance on the RMET increased with higher age, which was interpreted as the contribution of certain other variables reflecting cognitive skills (e.g., vocabulary) which continue to increase over the lifespan. Nonetheless, a meta-analysis of lab-based clinical investigations indicated large effect size on the RMET for the differentiation of people with schizophrenia or ASD from healthy controls (Hedges’g 0.73 and 0.81, respectively) [13].

With this in mind, it should be noted that while the RMET was developed to explore social understanding and interaction, the test has not been evaluated in an actual social interaction task, as it was typically examined only in single subject designs, i.e., testing one study participant at a time [7, 8]. However, the single-subject designs may lack ecological validity and have limited generalizability with respect to real, on-going, reciprocal social interactions. Social cognition during social interaction may principally differ from social cognition during social observation [19].

We identified one study which examined how TOM, as assessed by the RMET, can predict cooperative intentions based on video clips and photographs displaying people playing a variation of a Prisoner’s Dilemma game [1]. This study found no clear evidence for the relationship between ToM and cooperative intention recognition. However, based on the data from the study, it still remains unclear whether RMET is capable to predict actual performance when the subjects are involved in cooperative interaction. Recognition of cooperative intention may be fundamentally different from the actual action, as there may be no direct linear pathway from perception to action [20].

In the current study, we examined how well RMET predicts performance in healthy adults in a collaborative task requiring actual, real-time interpersonal coordination at the behavioral level. We used a collaborative task because everyday life requires coordinating our actions with other people, and mutual coordination play a fundamental role in human interactions. In a broader context, evolutionary psychologists study the origins of human behavior and cooperation with great interest because human societies are built on cooperation [21]. To achieve cooperation, participating individuals need to evaulate each other’s behavior for a common goal, which is accomplished through their ability for TOM.

TOM ability exhibits developmental changes, with many studies showing an inverse relationship between performance on the RMET and age, with most studies reporting decline (e.g [16–18]). This may be related to decline of the integration of information from multiple social sources [22] or to an impairment in extracting mental state information when social cues are limited in general (e.g., when the information is derived specifically from the eyes) [18]. Furthermore, prior findings indicate a decline with higher age regarding performance in multiple task monitoring and executive functions [23]. However, to our knowledge no study has examined the functional significance of these changes in a collaborative task during actual interaction between individuals. Thus, it remains to be determined whether an age related change in RMET is associated with a change in social interaction performance. Based on the previous findings which indicate a decline with higher age regarding performance in multiple task monitoring and executive functions, we expected lower performance in behavior synchronization in adults at a higher age.

Our first aim was to examine the predictive validity of RMET for the synchronous behavior of two interacting individuals during a reaction time task, which has been used to investigate social coordination [24, 25]. Our second aim was to investigate whether age-related changes in RMET performance are associated with changes in interpersonal coordination. Furthermore, the third aim was to examine whether an association between RMET performance and interpersonal coordination exists independent of various potentially important covariates, including the level of psychological distress and basic personality domains that have particular relevance during interpersonal coordination, including lack of attention and impulsive responding.

## Materials and methods

### Participants

Forty-eight healthy subjects (24 dyads, random pairs, unfamiliar to each other) participated in the study. Recruitment occurred using various channels, such as direct invitations, email announcements, and advertisements posted on university platforms and social media. Exclusion criteria were: any present or past psychiatric and neurological disorder, and a history of head injury with loss of consciousness. All 48 participants (24 dyads) were included in the study because they did not meet any exclusion criteria. Participants provided written informed consent before the study, in accordance with a protocol approved by the institutional review board and compliant with the Declaration of Helsinki. For the performance of the study and to form the dyads, individuals] were matched by age (+ 5 years).

### Materials

The Symptom Check List 90R, a 90-item self-report scale was used to measure the overall severity of psychological distress and psychopathology. The scale was designed to assess symptom severity in nine general domains of psychopathology in a broad spectrum of populations, ranging from non-patient healthy subjects to individuals with psychiatric disorders [26]. Internal consistency for the nine subscales is high, with Cronbach’s alpha coefficients from 0.77 to 0.90. The SCL-90-R has good convergent validity, correlating with other measures of psychological distress, such as the Beck Depression Inventory and the State-Trait Anxiety Inventory. The severity on the SCL-90R scale was assessed by the total score of the scale in order characterize the total symptom load on the scale, ranging for 0 to 360 points. Total score scale score measures general psychological distress with high reliability [27, 28].

For the characterization of lack of attention and impulsivity we adopted the two respective subscales of the Conners ADHD Scale’s adult version (CAARS; 66-item version) [29]: Inattention and Impulsivity. CAARS is a psychometrically sound scale demonstrating good test-retest reliability and internal-consistency (coefficient-alpha from 0.86 to 0.92 for its four factors, including with a Inattention, Hyperactivity, Impulsivity and Problems with Self-Concept) [30]. Analyses of large normative databases for the CAARS from various countries [31, 32] indicate substantial variation in severity along these symptom dimensions in normal populations. One study reported that nearly 60% of the general population have sub-threshold symptoms [33].

We used the Inattention and Impulsivity subscales since they capture behavioral manifestions of the personality traits such as low effortful control [34] and impulsiveness [35], which are potentially relevant to the task. These traits can interfere with cooperation by causing inattentiveness and impulsive responses. These can, respectively, lead to the omission of important salient signals, and to a sudden unpremeditated urge to react, thereby diminishing the proactive control for coordination.

The level of inattention and impulsivity was indexed, respectively, by the symptom severity on two psychopathological subscales of the CAARS scale: Inattention and Impulsivity. The Inattention subscale consists of 11 items, each of them scored on a 4-point scale between 0 and 3; therefore, the maximum total score of the subscale is 36 points. The Impulsivity subscale has 12 items, scoring between 0 and 3, resulting in a maximum total score of 33 points. A large, Ducth population-based study analyzing data from 4987 subjects showed that 28.5% of the general population sample had at least one inattention symptom and 46.1% have at least one hyperactivity symptom [36].

In order to characterize TOM ability, we applied the full set of 36 pictures from the RMET test battery. During the RMET testing, the subject is presented with the series of 36 images of the eye-region of the face of different actors and actresses, and is asked to choose which of four words best describes what the person in the images is thinking or feeling. During the test development for the RMET, the pictures were selected to represent complex mental states involving attribution of a belief or intention, instead of the universally recognized basic emotions (e.g., happy, sad, angry, afraid, and disgust; Supplemental material PART A). This also makes the RMET task more challenging, and increases the likelihood of obtaining a greater range of performance in a random sample of adults [7]. RMET’s psychometric properties have been widely investigated, and the test was validated in various settings. The internal consistency of the test was found to be acceptable, in two studies, Cronbach’s alpha was 0.605 [10] and 0.7 [37], respectively. It showed good convergent and discriminant validity [10]. RMET’s test-retest reliability has been demonstrated based on a one-year longitudinal study [38]. In three additional studies, the test-retest reliability of RMET, as measured by the intraclass-correlation coefficient were 0.60 [39], 0.92 [31], and 0.64 [40], respectively. The RMET test has been translated into Hungarian and validated [41]. The Hungarian version of the instrument applied in the current study is available publicly at the website of University of Cambridge Autism Research Centre [42]. The RMET test was administered separately to each of the two participants prior to the behavioral sessions. Based on pictures of pairs of eyes, presented in the RMET test, a decision should be made as to what complex emotions and mental states they express. The performance on the RMET is quantified as the number of correct responses during the 36 picture presentations. In the current study, the Theory of Mind ability was characterized by the proportion (%) of correct answers from the RMET test.

### Stimuli and procedure

To examine the synchronous behavior of two individuals during interpersonal coordination, we adopted a Go/NoGo reaction time task requiring response inhibition that we applied in our previous study [43]. The two participants who participated in an experimental session were seated side by side. Pictures from the International Affective Picture System were used as stimuli (see Supplemental material PART B for an illustration), presented in random sequence. Stimuli were shown centrally at every 1400ms for 800ms. A total of 486 pictures (in two sessions, 243 in each) were presented as stimuli by the Neurobehavioral Systems’ Presentation Software (negative, positive & neutral pictures with equal probability). The subjects were asked to push the button as soon as possible upon appearance of the stimulus pictures (Go trials); they were asked not to respond if a picture was repeated (NoGo trials). In the cooperative task we used, both subjects were asked not to compete, but collaborate with each other: to push the button simultaneously as soon as possible with the least possible number of errors. The two participants’ goal was to collect, together, as many correct answers as possible. If both participants responded to the Go stimulus within a preset time of 800 msec, the fixation cross preceding the next stimulus turned white, thereby giving a feedback signal; the cross turned red if the joint reaction was considered incorrect (if either participant fell outside the preset range or missed the response).

If none of them pressed the button in a NoGo trial (i.e., they both retained the response correctly), then their response was considered correct; if either of them pressed the button, the response was viewed as incorrect.

### Statistical analysis

We used the reaction times in msec for the individual Go stimuli as our primary measure to characterize interpersonal coordination (joint action). The reaction times were determined for each stimulus trial in each individual. We provided summary statistics at the level of the whole group and each individual. We also examined the intra-individual (within-subject) response time variability (ISV). Due to the high correlation of intraindividual response time variability and mean response time, the Coefficient of Variation (CV) is often used in the literature to characterize ISV [44], which we also adopted (CV) for the current study. We used the proportion of ommission errors (percent missed button presses for Go stimuli) and commission errors (the percent of button presses in the NoGo trials).

Synchronous behavior was characterized by the Pearson-correlation of participants’ responses in terms of RTs. Participants’ synchronous behavior was indexed by the correlation of the pairs of responses by the two participants. The correlation was computed for consequtive epochs of 20s duration to mitigate the potential effects of fatigue, habituation, and change in alertnes during the session, which can lead to an artifactual inflation of synchronization.

The statistical analysis was based on the random-regression, Hierarchical Linear Model (HLM). We adopted HLM because this methodology, as compared to traditional General Linear Model (GLM) approaches, is based on less restrictive assumptions and can efficiently exploits information on individual variability and sequential correlations among repeated measurements [45, 46]. Repeated measurement of correlation across the session between the reaction times of the pairs of participating subjects was used as the dependent variable in the HLM. A first order autoregressive correlation matrix was applied in the analysis to model the correlation structure between the consecutive epochs. Performance on the RMET test (i.e., proportion of correct recognition) served as independent variable. Covariates included in the analysis were: age, gender, SCL-90R total score, the CAARS Inattention and Impulsivity subscale scores, and behavioral measures such as the mean reaction time (RT) of participants and the Coefficient of Variation (CV) of RT’s variability. To characterize the effect sizes of the covariates in a comparable metric, we expressed the covariate values (Inattention, Impulsivity, RT, CV) in z-scores.

## Results

### Basic descriptive characteristics of the study sample

The basic demographic and clinical characteristics of the study group are summarized in Table 1. The proportion of female subjects was 65.0% in the sample, and the mean age was 32.9 (SD = 12.8) years. College/University degree was obtained by 25.0% of the subjects. The mean total score on the CAARS Inattention subscale was 8.7(SD = 6.1) points, which indicates that the study subjects scored in average at the lower quartile of the theoretical maximum (36 points) of the subscale (i.e., 24.2%=8.7/36). The data were similar to the CAARS Impulsivity subscale: the mean total score was 7.5(SD = 5.0) points, indicating that subjects also scored in average at the lower quartile of the theoretical maximum (33 points) of this subscale (i.e., 22.7%=7.5/33). The mean total score SCL-90R was 25.04(SD = 22.2). The RT averaged 371.4(SD = 55.7) msec, with a variability of approximately 22% in terms of the Coefficient of Variation, and no significant difference among positive, neutral and negative pictures in the ANOVA analysis (*p* = 0.16). In the average, study subjects missed the stimulus pictures in 2.2%(SD = 0.53%) (omission errors) of the presentations and they had an average commission error rate of 12.4%(SD = 2.1%). The performance (accuracy) on the RMET test was 71%(SD = 10.0%).

**Table 1:** Basic descriptive characteristics of the study sample

Characteristics	Study Sample (*n* = 48) 24 pairs	
**Categorical variables**	N	%
F/M	31/17	65/35
Education level, without college degree/with college degree	36/12	75/25
**Continuous variables**	**Mean**	**SD**
Age, y	32.9	12.8
Years of education	12.9	2.2
**CAARS** ^a^		
Inattention	8.7	6.1
Impulsivity	7.5	5.0
**SCL-90R** ^b^	25.04	22.2
RMET^c^ (recognition proportion%,SD)	71	10
Reaction time(msec)	371.4	55.7
Intra-Individual Variability, Coefficient of Variation (CV,%)	22.1	11.2
Omission errors(%)	2.2	0.53
Commission errors(%)	12.4	2.1

^a^ CAARS = Conners Adult ADHD Rating Scale

^b^ SCL-90R = The Symptom Checklist-90R, total score

^c^ RMET = Reading the Mind in the Eyes Test

### Behavioral response synchronization as a function of performance on the RMET

**Fig. 1:**
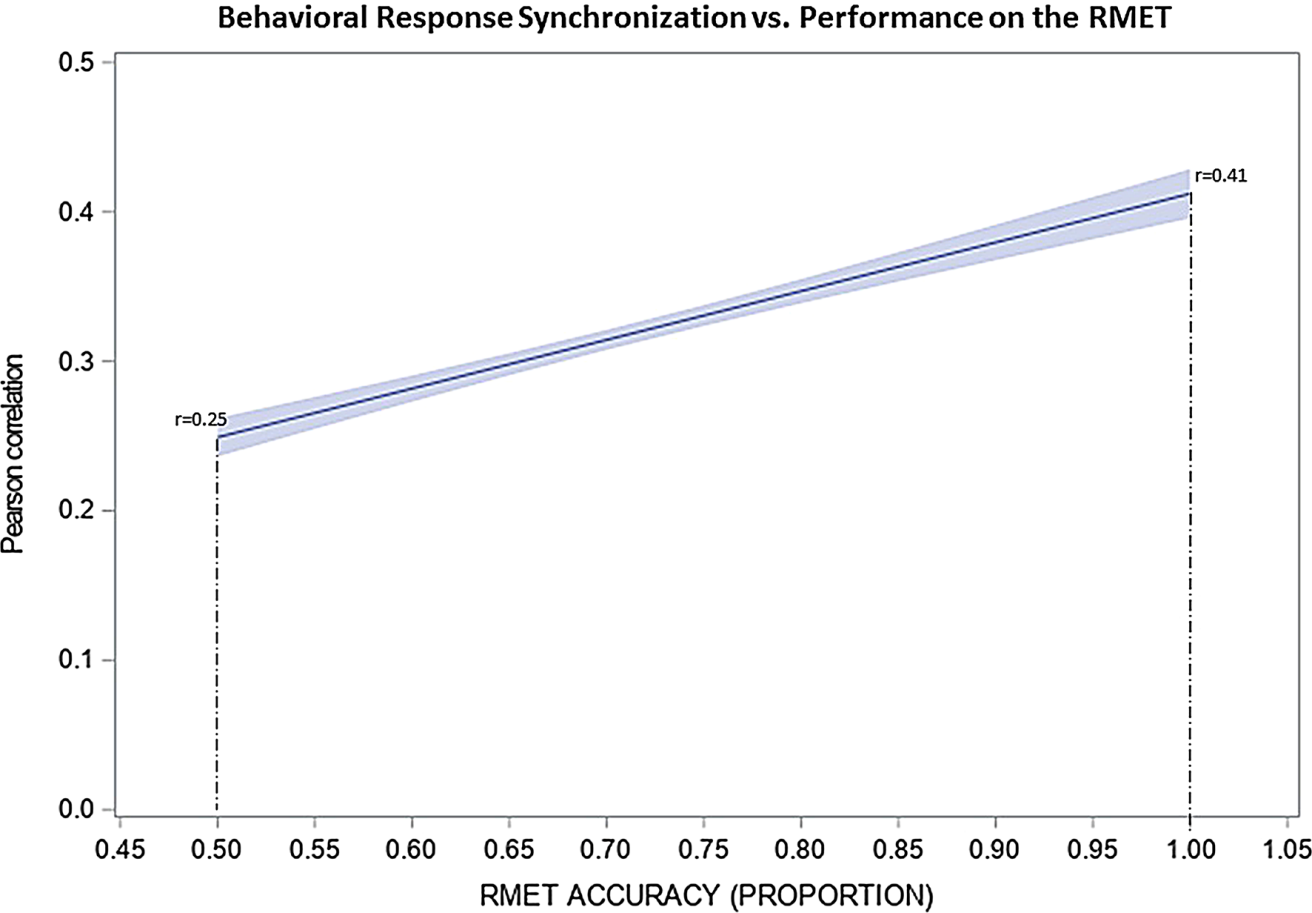
Synchronization of behavioral responses (i.e., RTs) between the interacting pairs as indexed by the Pearson correlation. On the vertical axis Pearson-r is shown as a function of the correct recognition rate (horizontal axis) on the RMET. The figure depicts the regression line for the relationship. The shaded area around the line indicates the 95% confidence intervals. The vertical dashed lines indicate regression estimates corresponding to a correct recognition rate of 50% and 100% on the RMET, respectively

We found a significant positive association between the synchronization of the behavioral responses (i.e., RTs) of the participating pairs and the performance measured by the RMET (F = 153.34,*p* < 0.0001) (Fig. 1). As shown by the Figure, with good performance (100% correct recognition), the behavioral response synchronization was significantly higher (Pearson correlation, *r* = 0.41) than with poor performance (50% correct recognition, *r* = 0.25).

### Behavioral response synchronization vs. performance on the RMET in relation to age

**Fig. 2:**
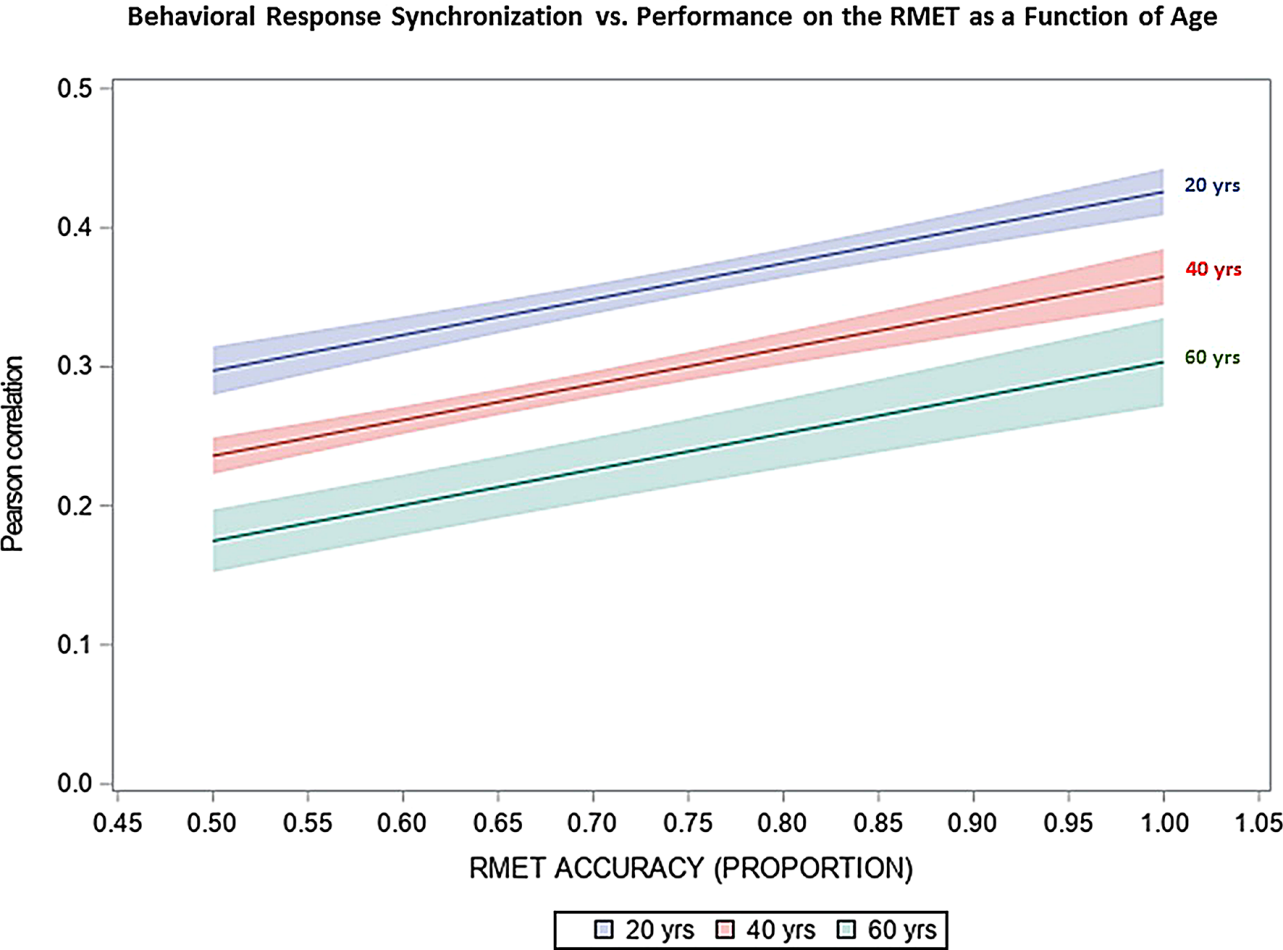
Association between behavioral response synchronization and performance on the RMET. Our findings indicate that the positive relationship between behavioral response synchronization and performance on the RMET remains significant after inclusion of age as covariate in the analyses (adjusted F = 52.47,*p* < 0.0001). Furthermore, we found that higher age was associated with lower synchronization (F = 63.14,*p* < 0.0001). To illustrate the covariate effects of age based on the regression LSMEAN estimates, the figure depicts the association between behavioral synchronization and RMET performance in the form of regression lines computed for 20, 40 and 60 years of age

Our further analyses showed that the performance on the RMET was inversely related to age (F = 5.79,*p* = 0.022), whereas it was not associated with gender (F = 0.001,*p* = 0.979) or education level (F = 0.18,*p* = 0.71). Inclusion of these variables as additional independent variables in HLM model did not change the significance of the association between behavioral response synchronization and performance on the RMET (adjusted F = 52.47,*p* < 0.0001). However, the results of these analyses revealed that behavior synchronization was significantly associated with age (F = 63.14,*p* < 0.0001), but not with gender or education level (*p* > 0.1 for both variables). Specificaly, our findings showed that higher age was linked to lower synchronization, without a significant interaction (*p* > 0.1) between the two variables (Fig. 2).

### Subsidiary analyses

We used the total score on the SCL-90R scale to characterize the overall severity of psychological distress. After including this variable as a covariate in the main analysis, our results showed that while the RMET’s effects on behavior synchronization kept statistical significance (F = 52.56, *p* < 0.0001), the effect of SCL-90R total score was not significant (*p* > 0.05). After the inclusion of the CAARS Inattention and Impulsivity scores into the main analysis model, the results showed essentially no change with respect to RMET’s contribution to behavioral synchronization (F = 65.13,*p* < 0.0001).

**Fig. 3:**
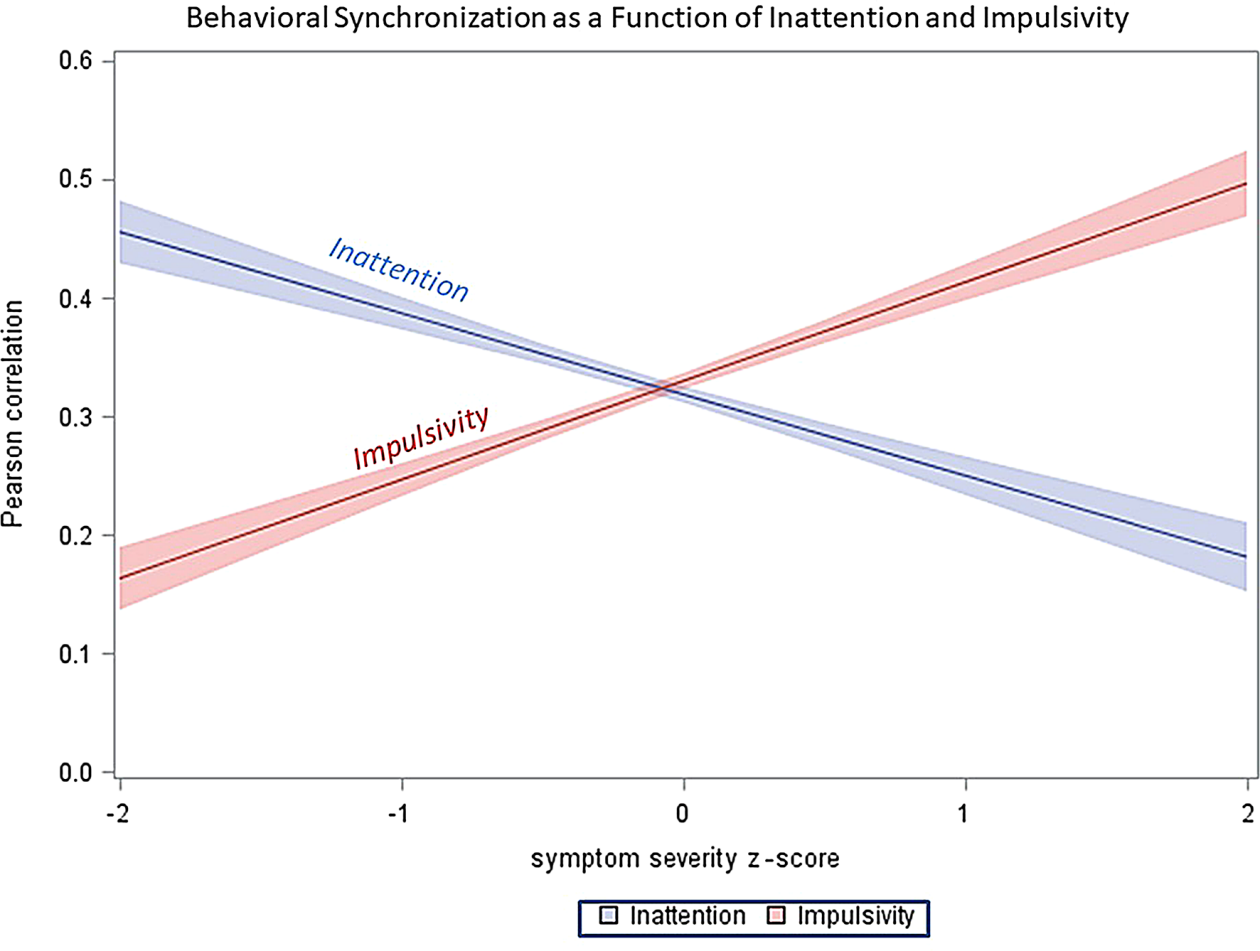
Behavioral Synchronization as a Function of Inattention and Impulsivity subscale scores of the CAARS scale. Please note that Inattention and Impulsivity scores are expressed in z-score units to depict the two variables in the same metrics

Importantly, however, the two covariates, inattention and impulsivity, had a significant association with behavioral synchronization. Specifically, inattention was negatively related to synchronization (t =-10.06,*p* < 0.0001), i.e., higher level of inattention was accompanied by lower levels of synchronization. By contrast, impulsivity was positively associated with behavioral synchronization (t = 12.59,*p* < 0.0001), i.e., higher level of impulsivity was accompanied by greater synchronization (Fig. 3).

**Fig. 4:**
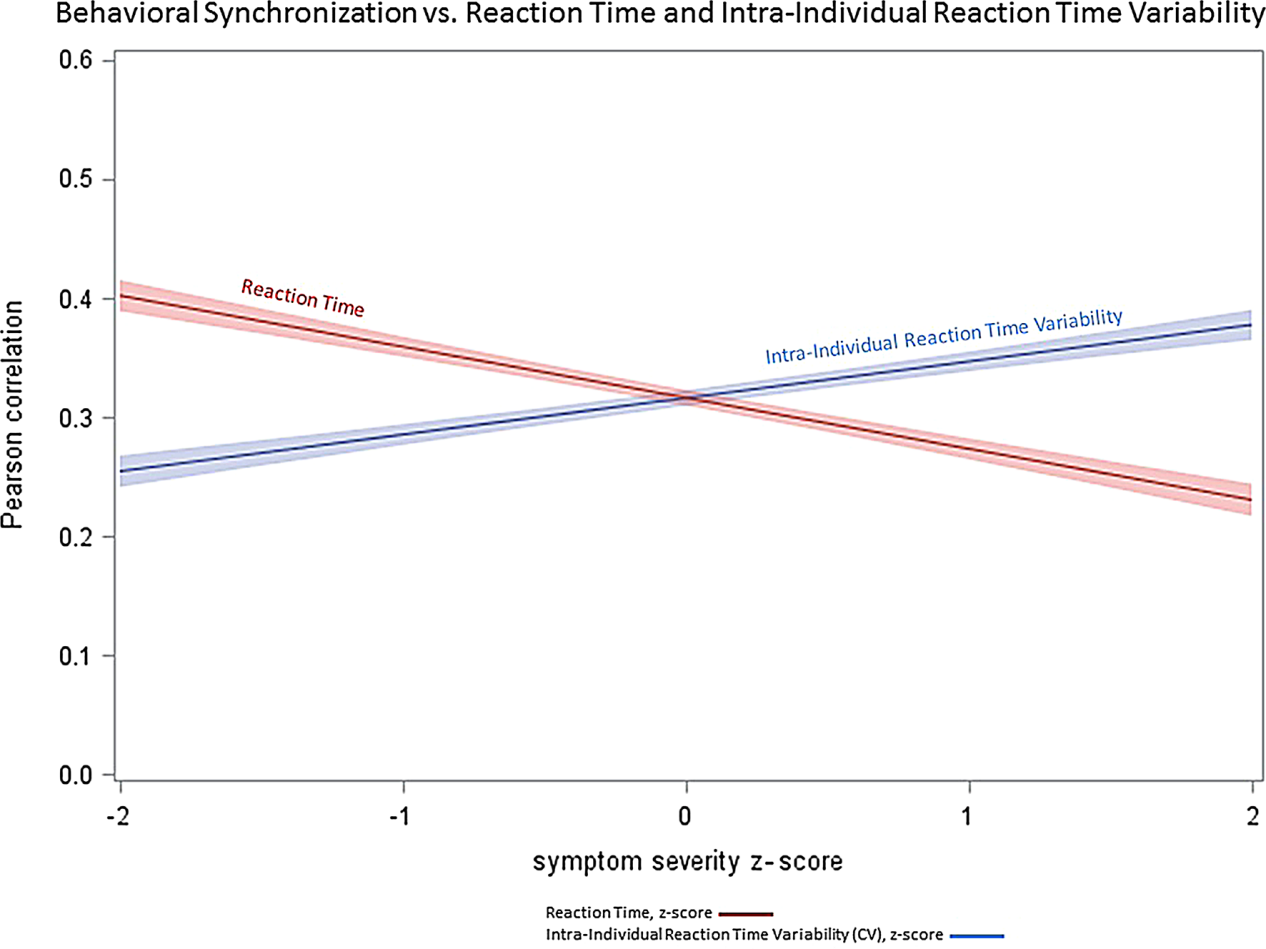
Behavioral synchronization as a function of reaction time (RT) and reaction time variability (Coefficient of Variation, CV). Please note that RT and CV are expressed in z-score units to depict the two variables in the same metrics

In further subsidiary analyses we investigated whether the speed of responding and its variability influenced behavioral synchronization. We found that synchronization increased significantly with shorter reaction times (F = 210.61,*p* < 0.0001). Furthermore, as shown by Fig. 4, the synchronization also increased significantly with greater reaction time variability (CV) (F = 120.0,*p* < 0.0001). We note that the inclusion of these two covariates in the analyses did not alter the significance of the association between RMET performance and behavioral synchronization (*p* < 0.05, after adjusment).

## Discussion

The RMET test was developed to explore social understanding [6]. The test is widely used in research into the theory of consciousness both in healthy people and among psychiatric patients including individuals with schizophrenia, ADHD and borderline personality disorder [47–49]. Furthermore, it is often applied as a diagnostic aid for well-functioning people with ASD [7, 50].

The RMET test has been validated in a variety of single-subject designs [51, 52], but so far its validity has not been investigated in multiple-subject design, during actual interpersonal interactions. To the best of our knowledge, the current study is the first to investigate the predictive validity of RMET, i.e., to examine how the test predicts synchronous behavior in two interacting subjects during interpersonal coordination. Our results provide supportive data for RMET’s capacity to predict interpersonal coordination; specifically, they show that the higher the performance on the RMET, the higher the behavioral synchronization between the participating subjects. This relationship was specific to the RMET because it remained significant after correction for the covariates.

We assessed the predictive capacity of RMET, as a measure of social understanding, in association with the covariates which included demographical, psychopathological and behavioral data. With respect to basic demographic variables we investigated age, gender and education level. While gender and education level did not show a significant relationship, the subjects’ age had a strong main effect for synchronization; the higher the age, the lower the synchronization. These results are consistent with findings [23] that, due to reduced executive control functions, the balancing among multiple goals with conflicting costs (such as minimizing error while maintaining synchronization, as required in our study) is more challenging for older adults. Additionally, our data showed that RMET and age did not show an interaction, they exert their effects independently in an additive way. Our finding with respect to the age-related decline in performance on the RMET is consistent with the bulk of the data in the literature [16, 18] but in view of the opposite findings from a large internet-based study [15] the evidence from the various sources requires further investigation. Furthermore, as our study had a cross-sectional design, the question of whether age-related changes in RMET are associated with changes in social interaction performance needs future studies using a longitudinal design.

While adjustment for inattention and impulsivity did not influence relationship with RMET, both of these variables had a strong and independent relationship with interpersonal synchronization. However, the relationship was opposite for inattention and impulsivity. In particular, higher level of inattention was accompanied by lower level of synchronization. By contrast, impulsivity was positively associated with behavioral synchronization: greater level of impulsivity was accompanied by greater synchronization.

Whereas the association of interpersonal coordination with inattention has face validity (since the less one can focus on the task, the lower the expected performance), the association with impulsivity appears counterintuitive at the first glance. However, one has to take into consideration that impulsivity is increasingly being seen as a multidimensional construct [53]. Specifically, while impulsivity was frequently conceived as a maladaptive trait (i.e., impulse to act without thinking) and has been linked to a variety of mental disorders [54–56], emerging evidence indicates that it can be beneficial in certain contexts which require quick adaptation, especially when rapid responding and attentional focusing is required [53]. The experimental task we used represents such a situation; it requires a quick reaction and adjustment, which may be behind our finding of the positive association between impulsivity and behavioral coordination. Moreover, it is worth noting that the cooperative go/no-go task provides a useful framework for evaluating cooperative success, as it encompasses critical aspects of coordinated behavior, such as shared goals and interpersonal synchronization. Our findings regarding the behavioral data indicated that both the reaction time and its variability, as indexed by the CV, showed a significant relationship with synchronization. Specifically, shorter reaction time and higher response variability were associated with better behavioral synchronization. We think that shorter reaction time and greater response variability are likely to be associated with better adaptability, which leads to greater flexibility to adjust to the partner’s responses. These behavioral findings are therefore consistent with those pertaining to impulsivity and, taken together, highlight the role of adaptability in the social coordination.

### Limitations

In our study, the instruction for the subjects was to respond simultaneously as soon as possible with the least possible number of errors, regardless of the emotional content. Nonetheless, the emotional valence of the pictures could have interfered with task performance, which would require further investigation. We used a stimulus-response paradigm that required fast and stimulus driven responses from the participants with rapid decision making. Although the cooperative go/no-go paradigm serves as a useful tool for assessing cooperative success, real-world cooperation is influenced by various factors (e.g., social norms) that differ across contexts (e.g., workplace, family). Thus, the task may not fully capture the unique challenges in each setting. With respect to inattention and impulsivity, we would like to emphasize that these trait characteristics represent a dimensional measure for subjects screened for neurological or psychiatric disorders, which were exclusion criteria from our study. Therefore, it is unclear whether our findings can be dimensionally extrapolated to patients with such disorders. As we adjusted for the potential confounding of age, gender and education level, it is not likely that our principal findings with respect RMET’s predictive capacity for interpersonal coordination were confounded by these factors. Nonetheless, future studies are needed with a larger sample size recruited by using a different selection strategy, and an implementation of a comprehensive evaluation of demographic and socio-cultural effects. It is also important to note that a study with a dyadic design is difficult to carry out due to its underlying requirements in terms of logistics and organization, which limits the sample size achievable with the design. Finally, it is conceivable that the moment-to-moment fluctuations in the stress levels of the subjects (e.g., due to misjudgements by the dyads) could have influenced performance, which should be addressed in the future. In addition, various other factors such as performance anxiety and /or level of perceived stress, error frustration, presence of alexithymia symptoms and poor neuropsychological abilities can interfere with the task performance. Therefore, the impact of these variables needs to be examined future studies.

## Conclusions

Our results support the predictive validity of RMET, as a measure of mentalization ability, with respect to actual interpersonal collaboration. With respect to our first objective, the results indicated that the synchronization of the behavioral responses (i.e., RTs) between the interacting pairs and their mentalization ability, as measured by the RMET, showed significant association: with good performance on the RMET, the behavioral response synchronization was higher than with poor performance. As to the second objective, we found that higher age was inversely related to behavioral synchronization. With respect to the moderating effect of additional covariates (third objective), our findings indicate that their inclusion of additional covariates in the analyses did not alter the significance of the association between RMET performance and behavioral synchronization.

The delination of the underpinnings of social interactions is of great importance both in the investigation of everyday collaborations among healthy individuals and in the identification of social interaction problems in various psychiatric disorders. Thus, the demonstration of utility of RMET in predicting functioning during actual interpersonal collaboration has important inplications for its clinical use, and can extend the range of potential applications.

## Electronic supplementary material

Below is the link to the electronic supplementary material.

Supplementary Material 1: **Part/A**: Illustration of the “Reading the Mind in the Eyes” (RMET) test. **Part/B**: Example stimuli from the 243 pictures of the International Affective Picture Set (IAPS) that were presented in a session in the current study.

## Data Availability

The datasets analyzed during the current study are not publicly available due to privacy restrictions, but are available from the corresponding author upon reasonable request.

## Abbreviations

RMET: Reading the Mind in the Eyes Test
RTs: Reaction times
RT: Reaction time
TOM: Theory of Mind
ASD: Autism spectrum disorder
ISV: Intra-individual (within-subject) response time variability.
CV: Coefficient of Variation.
HLM: Hierarchical Linear Model.
GLM: General Linear Model.

## References

[1] Sylwester K, Lyons M, Buchanan C, Nettle D, Roberts G. The role of Theory of Mind in assessing cooperative intentions. Pers Individ Dif. 2012;52:113–7.

[2] Davies M, Stone T. Synthesis: Psychological understanding and social skills. In: Davies M, Stone T, editors. Individual Differences in Theory of Mind: Implications for Typical and Atypical Development. Hove: Psychology Press; 2003. p. 303-50.

[3] Paal T, Bereczkei T. Punishment as a means of competition: implications for strong reciprocity theory. PLoS One. 2015;10:e0120394. 25811464 10.1371/journal.pone.0120394 PMC4374862

[4] Cloninger CR, Bayon C, Svrakic DM. Measurement of temperament and character in mood disorders: a model of fundamental states as personality types. J Affect Disord. 1998;51:21–32. 9879800 10.1016/s0165-0327(98)00153-0

[5] Fonagy P, Gergely G, Jurist EL, Target M. Affect regulation, mentalization, and the development of the self. New York: Other; 2002.

[6] Baron-Cohen S, Jolliffe T, Mortimore C, Robertson M. Another advanced test of theory of mind: evidence from very high functioning adults with autism or asperger syndrome. J Child PsycholPsychiatry. 1997;38:813–22. 10.1111/j.1469-7610.1997.tb01599.x 9363580

[7] Baron-Cohen S, Wheelwright S, Hill J, Raste Y, Plumb I. The reading the mind in the eyes test revised version: a study with normal adults, and adults with Asperger syndrome or high-functioning autism.J. Child Psychol Psychiatry. 2001;42:241–51. 11280420

[8] Kirkland RA, Peterson E, Baker CA, Miller S, Pulos S. Meta-analysis reveals adult female superiority in reading the mind in the eyes Test.North American. J Psychol. 2013;15:121–46.

[9] Oakley BFM, Brewer R, Bird G, Catmur C. Theory of mind is not theory of emotion: a cautionary note on the reading the mind in the eyes test. J Abnorm Psychol. 2016;125:818–23. 27505409 10.1037/abn0000182 PMC4976760

[10] Vellante M, Baron-Cohen S, Melis M, Marrone M, Petretto DR, Masala C, Preti A. The reading the mind in the eyes test: systematic review of psychometric properties and a validation study in Italy. Cogn Neuropsychiatry. 2013;18:326–54. 23106125 10.1080/13546805.2012.721728 PMC6345369

[11] Rutherford HJ, Wareham JD, Vrouva I, Mayes LC, Fonagy P, Potenza MN. Sex differences moderate the relationship between adolescent language and mentalization. Personality Disorders. 2012;3(4):393–405. 22800178 10.1037/a0028938 PMC3691855

[12] Frick C, Lang S, Kotchoubey B, Sieswerda S, Dinu-Biringer R, Berger M, Veser S, Essig M, Barnow S. (2012). Hypersensitivity in borderline personality disorder during mindreading. PLoS ONE, 7(8), e41650. 10.1371/journal.pone.0041650 PMC3411703 22870240

[13] Chung YS, Barch D, Strube M. A meta-analysis of mentalizing impairments in adults with schizophrenia and autism spectrum disorder. Schizophr Bull. 2014;40:602–16. 23686020 10.1093/schbul/sbt048 PMC3984506

[14] Castelli F, Frith C, Happe F, Frith U. Autism, Asperger syndrome and brain mechanisms for the attribution of mental states to animated shapes. Brain. 2002;125:1839–49. 12135974 10.1093/brain/awf189

[15] Feder-Dodell D, Ressler J, Kerry, Germine T, Laura. Social cognition or social class and culture? On the interpretation of differences in social cognitive performance. Psychol Med. 2018;52:133–45. 10.1017/S003329171800404X 30616706

[16] Chander RJ, Grainger SA, Crawford JD, Mather KA, Numbers K, Cleary R, Kochan NA, Brodaty H, Henry JD, Sachdev PS. Development of a short-form version of the reading the mind in the eyes test for assessing theory of mind in older adults. Int JGeriatr Psychiatry. 2020;35:1322–30. 32584445 10.1002/gps.5369

[17] Franklin RG Jr., Zebrowitz LA. Aging-related changes in Decoding negative complex Mental States from Faces. Exp Aging Res. 2016;42:471–8. 27749208 10.1080/0361073X.2016.1224667 PMC5189905

[18] Kynast J, Quinque EM, Polyakova M, Luck T, Riedel-Heller SG, Baron-Cohen S, et al. Mindreading from the eyes declines with aging-evidence from 1,603 subjects. Front Aging Neurosci. 2020;12:55041. 10.3389/fnagi.2020.550416 PMC7656776 33192452

[19] Redcay E, Schilbach L. Using second-person neuroscience to elucidate the mechanisms of social interaction. Nat Rev Neurosci. 2021;20:495–505. 10.1038/s41583-019-0179-4 PMC6997943 31138910

[20] Pfeifer R, Scheier C. From perception to action: The right direction? In: Gaussier P, Nicoud J-D, editors. From Perception to Action. Los Alamitos (CA): IEEE Computer Society Press; 1994. p. 1-11.

[21] Fábio Portela Lopes Almeida. The evolutionary origins of human cooperation and its implications for legal theory. RevistaDireito GV. 2013;9:243–68.

[22] Hunter EM, Phillips LH, MacPherson SE. Effects of age on cross-modal emotion perception. Psychol Aging. 2010;25:779–87. 10.1037/ a0020528. 21186914 10.1037/a0020528

[23] Todorov I, Del MF, Mantyla T. Age-related differences in multiple task monitoring. PLoS One. 2014;9:e107619. 25215609 10.1371/journal.pone.0107619 PMC4162647

[24] Kruppa JA, Reindl V, Gerloff C, Oberwelland WE, Prinz J, Herpertz-Dahlmann B, Konrad K, Schulte-Ruther M. Brain and motor synchrony in children and adolescents with ASD-a fNIRS hyperscanning study.Soc. CognAffect. Volume 16. Neurosci; 2021. pp. 103–16. 10.1093/scan/nsaa092 PMC7812623 32685971

[25] Wang Q, Han Z, Hu X, Feng S, Wang H, Liu T, Yi L. Autism symptoms modulate interpersonal neural synchronization in children with Autism Spectrum Disorder in Cooperative interactions. Brain Topogr. 2020;33:112–22. 31560088 10.1007/s10548-019-00731-x

[26] Derogatis LR, Cleary PA. Factorial invariance across gender for the primary symptom dimensions of the SCL-90.Br. J Soc Clin Psychol. 1977;16:347–56. 10.1111/j.2044-8260.1977.tb00241.x 588890

[27] Derogatis LR. The SCL-90–R^®^: administration, scoring and procedures manual. 3rd ed. Minneapolis, MN: National Computer Systems; 1994.

[28] Smits IAM, Timmerman ME, Barelds DPH, Meijer RR. The Dutch Symptom Checklist-90-Revised: is the use of the subscales justified? Eur J Psychol Assess. 2015;31(4):263–71.

[29] Conners CK. Clinical use of rating scales in diagnosis and treatment of attention-deficit/hyperactivity disorder. Pediatr Clin North Am. 1999;46(5):857–70. 10.1016/s0031-3955(05)70159-0 10570692

[30] Erhardt D, Epstein JN, Conners CK, Parker JDA, Sitarenios G. Self-ratings of ADHD symptomas in auts II: reliability, validity, and diagnostic sensitivity. J Atten Disord. 1999;3: 153–8.

[31] Christiansen H, Kis B, Hirsch O, Philipsen A, Henneck M, Panczuk A, Pietrowsky R, Hebebrand J, Schimmelmann BG. German validation of the Conners adult ADHD rating scales-self-report(CAARS-S) I: factor structure and normative data. Eur Psychiatry. 2011;26:100–7. 20619613 10.1016/j.eurpsy.2009.12.024

[32] Christiansen H, Hirsch O, Philipsen A, Oades RD, Matthies S, Hebebrand J, Ueckermann J, Abdel-Hamid M, Kraemer M, Wiltfang J, Graf E, Colla M, Sobanski E, Alm B, Rosler M, Jacob C, Jans T, Huss M, Schimmelmann BG, Kis B. German validation of the conners adult ADHD rating scale-self-report: confirmation of factor structure in a large sample of participants with ADHD. J Atten Disord. 2013;17:690–8. 22441889 10.1177/1087054711435680

[33] Arcos-Burgos M, Acosta MT. Tuning major gene variants conditioning human behavior: the anachronism of ADHD. Curr OpinGenet Dev. 2007;17:234–8. 10.1016/j.gde.2007.04.011 17467976

[34] Nigg JT. Temperament and developmental psychopathology. J Child Psychol Psychiatry Allied Discip. 2006;47(3–4):395–422. 10.1111/j.1469-7610.2006.01612.x 16492265

[35] Christiansen H, Kis B, Hirsch O, Matthies S, Hebebrand J, Uekermann J, Abdel-Hamid M, Kraemer M, Wiltfang J, Graf E, Colla M, Sobanski E, Alm B, Rösler M, Jacob C, Jans T, Huss M, Schimmelmann BG, Philipsen A. German validation of the Conners adult ADHD rating scales (CAARS) II: reliability, validity, diagnostic sensitivity and specificity. Eur Psychiatry: J Association Eur Psychiatrists. 2012;27(5):321–8. 10.1016/j.eurpsy.2010.12.010 21392946

[36] Li T, Mota NR, Galesloot TE, Bralten J, Buitelaar JK, IntHout J, AriasVasquez A, Franke B. ADHD symptoms in the adult general population are associated with factors linked to ADHD in adult patients.Eur. Neuropsychopharmacol. 2019;29:1117–26. 10.1016/j.euroneuro.2019.07.136 31378654

[37] Charernboon T, Lerthattasilp T. The reading the mind in the eyes test: validity and reliability of the Thai Version. CognBehavNeurol. 2017;30:98–101. 10.1097/WNN.0000000000000130 28926417

[38] Fernandez-Abascal EG, Cabello R, Fernandez-Berrocal P, Baron-Cohen S. Test-retest reliability of the ‘Reading the mind in the eyes’ test: a one-year follow-up study. Mol Autism. 2013;4:33. 24020728 10.1186/2040-2392-4-33 PMC3848772

[39] Hallerback MU, Lugnegard T, Hjarthag F, Gillberg C. The reading the mind in the eyes test: test-retest reliability of a Swedish version. Cogn Neuropsychiatry. 2009;14:127–43. 19370436 10.1080/13546800902901518

[40] Chakrabarty M, Dasgupta G, Acharya R, Chatterjee SS, Guha P, Belmonte MK, Bhattacharya K. Validation of revised reading the mind in the eyes test in the Indian (Bengali) population: a preliminary study. Indian JPsychiatry. 2021;63:74–9. 34083824 10.4103/psychiatry.IndianJPsychiatry_967_20 PMC8106414

[41] Ivády RE, Takács B, Pléh C. Theory of Mind and acquisition of foreign language - real connection or urban Legend? (In Hungarian). (Tudatelmélet és Idegennyelv elsajátítás - valódi kapcsolat vagy városi legenda?) In: Tudat és Elme (Consciousness and Mind). pp. 59–74. Eds. Kampis G & Mund K. Publisher: Typotex, Budapest, 2007.

[42] https://www.autismresearchcentre.com/tests/eyes-test-adult/

[43] Czobor P, Kakuszi B, Németh K, Balogh L, Papp S, Tombor L, Bitter I. Electrophysiological indices of aberrant error-processing in adults with ADHD: a new region of interest. Brain Imaging Behav. 2017;11:1616–28. 27752922 10.1007/s11682-016-9610-x

[44] Suskauer SJ, Simmonds DJ, Caffo BS, Denckla MB, Pekar JJ, Mostofsky SH. fMRI of intrasubject variability in ADHD: anomalous premotor activity with prefrontal compensation. J Am Acad Child Adolesc Psychiatry. 2008;47:1141–50. 18724253 10.1097/CHI.0b013e3181825b1f PMC3932630

[45] Bryk AS, Raudenbush SW. Application of hierarchical linear models to assessing change. Psychol Bull. 1987;101(1):147–58.

[46] Bryk AS, Raudenbush SW. Hierarchical linear models: applications and data analysis methods. Sage Publications, Inc.; 1992.

[47] Aydin O, Balikci K, Sonmez I, Unal-Aydin P, Spada MM. Examining the roles of cognitive flexibility, emotion recognition, and metacognitions in adult attention deficit and hyperactivity disorder with predominantly inattentive presentation. Clin Psychol Psychother. 2021;29(2):542–53. 10.1002/cpp.2645 34272785

[48] Cyrkot T, Szczepanowski R, Jankowiak-Siuda K, Gaweda L, Cichon E. Mindreading and metacognition patterns in patients with borderline personality disorder: experimental study. Eur ArchPsychiatry Clin Neurosci. 2021;271:1159–68. 10.1007/s00406-020-01227-7 PMC8354944 33459868

[49] Navarra-Ventura G, Vicent-Gil M, Serra-Blasco M, Massons C, Crosas JM, Cobo J, et al. Group and sex differences in social cognition in bipolar disorder, schizophrenia/schizoaffective disorder and healthy people. Compr Psychiatry. 2021;109:15225. 10.1016/j.comppsych.2021.152258 34252633

[50] Moriuchi JM, Klin A, Jones W. Mechanisms of diminished attention to eyes in Autism. Am J Psychiatry. 2017;174:26–35. 27855484 10.1176/appi.ajp.2016.15091222 PMC5842709

[51] Oliver LD, Moxon-Emre I, Lai MC, Grennan L, Voineskos AN, Ameis SH. Social Cognitive performance in Schizophrenia Spectrum disorders compared with autism spectrum disorder: a systematic review, Meta-analysis, and Meta-regression. JAMA Psychiatry. 2021;78:281–92. 33291141 10.1001/jamapsychiatry.2020.3908 PMC7724568

[52] Penuelas-Calvo I, Sareen A, Sevilla-Llewellyn-Jones J, Fernandez-Berrocal P. The reading the mind in the eyes test in Autism-Spectrum disorders comparison with healthy controls:a systematic review and Meta-analysis. J Autism Dev Disord. 2019;49:1048–61. 30406435 10.1007/s10803-018-3814-4

[53] Toschi C, Hervig ME, Moazen P, Parker MG, Dalley JW, Gether U, et al. Adaptive aspects of impulsivity and interactions with effects of catecholaminergic agents in the 5-choice serial reaction time task: implications for ADHD. Psychopharmacology(Berl). 2021;238:2601–15. 10.1007/s00213-021-05883-y PMC8373759 34104987

[54] Baribeau DA, Dupuis A, Paton TA, Hammill C, Scherer SW, Schachar RJ, et al. Structural neuroimaging correlates of social deficits are similar in autism spectrum disorder and attention-deficit/hyperactivity disorder: analysis from the POND Network. Transl Psychiatry. 2019;9:72. 10.1038/s41398-019-0382-0 PMC6361977 30718456

[55] Tay SA, Hulbert CA, Jackson HJ, Chanen AM. Affective and cognitive theory of mind abilities in youth with borderline personality disorder or major depressive disorder. Psychiatry Res. 2017;255:405–11. 28667928 10.1016/j.psychres.2017.06.016

[56] Zabihzadeh A, Maleki G, Richman MJ, Hatami A, Alimardani Z, Heidari M. Affective and cognitive theory of mind in borderline personality disorder: the role of comorbid depression. PsychiatryRes. 2017;257:144–9. 10.1016/j.psychres.2017.07.034 28755605

