# Supplementary material for: Behavioral synchronization of two individuals during cooperative interaction: the role of the mentalization ability as measured by the reading the mind in the eyes test
Source: BMC Psychol. 2025 Feb 16;13:126. doi: 10.1186/s40359-025-02457-x (PMC11830189; doi:10.1186/s40359-025-02457-x)
Supplement: Supplementary file 1 — Supplementary Material 1: Part/A: Illustration of the “Reading the Mind in the Eyes” (RMET) test. Part/B: Example stimuli from the 243 pictures of the International Affective Picture Set (IAPS) that were presented in a session in the current study. [file 40359_2025_2457_MOESM1_ESM.docx]

**Online Supplementary Material**

**Part/A: Illustration of the „Reading the Mind in the Eyes” (RMET) test, using two example stimuli from the test**


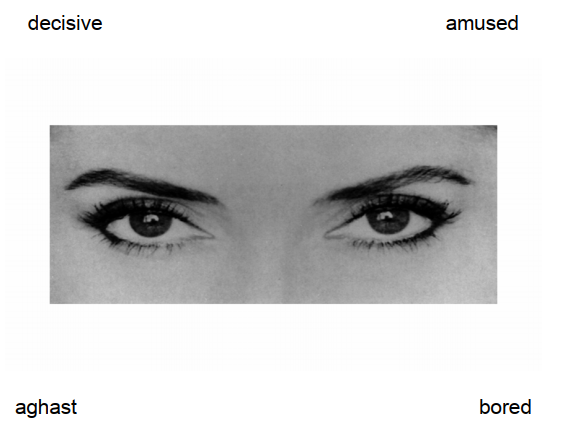
**
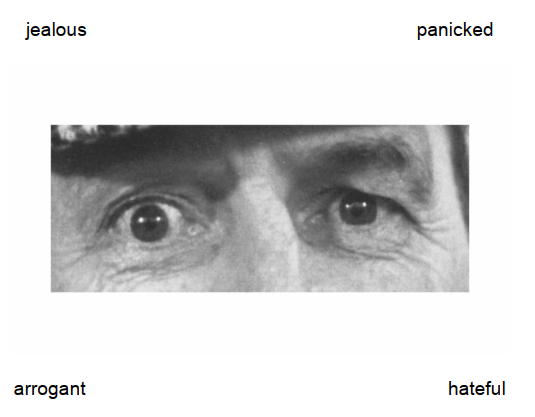
**

The RMET test is a measure mentalization, including social cognition and empathy. Participants have to select one complex mental state among four possibilities that they consider that best describes the emotion represented by the picture. The above pictures illustrate 2 of the 36 images with the layout of the test sheets that are shown to the subjects.

The full set of RMET stimuli are published and freely available online at <https://www.autismresearchcentre.com/arc_tests>). In the current study, the full set of the total of 36 pictures are shown to the subjects. *(Baron-Cohen, S. et al.(2001). The "Reading the Mind in the Eyes" Test revised version: a study with normal adults, and adults with Asperger syndrome or high-functioning autism.J. Child Psychol. Psychiatry 42,241-251)*

**Part/B: Example stimuli from the 243 pictures of the International Affective Picture Set (IAPS) that were presented in a session in the current study.**


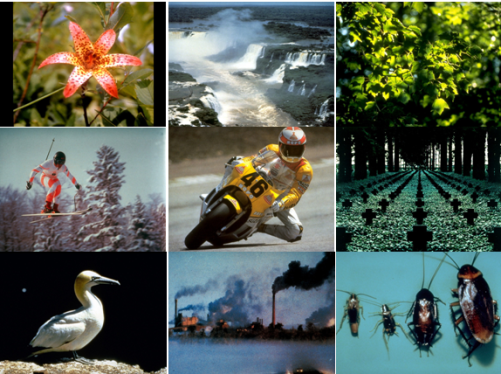


IAPS is an internationally available and standardized set of stimuli with varied emotional content designed to examine attentional and emotional processes. The above images show nine example stimuli from the set. In our study, subjects were asked to push a button as soon as possible upon appearance of the stimulus pictures (Go trial); they were, however, asked to withhold response in case a picture was repeated (NoGo trials).(*Lang P.J.et al(2008). International affective picture system (IAPS): Affective ratings of pictures and instruction manual. Techical Report A-8. University of Florida: Gainesville, FL.; Lang P.J et al. (2005). International affective picture system (IAPS):Affective ratings of pictures and instruction manual. Technical Report A-6. Gainesville:University of Florida.*
